# 2D-Shear Wave Elastography in the Evaluation of Parathyroid Lesions in Patients with Hyperparathyroidism

**DOI:** 10.1155/2017/9092120

**Published:** 2017-08-06

**Authors:** Ioana Golu, Ioan Sporea, Lavinia Moleriu, Anca Tudor, Marioara Cornianu, Adrian Vlad, Romulus Timar, Melania Balas, Daniela Amzar, Mihaela Vlad

**Affiliations:** ^1^Department of Endocrinology, “Victor Babes” University of Medicine and Pharmacy, Timisoara, Romania; ^2^Elastography Center, “Victor Babes” University of Medicine and Pharmacy, Timisoara, Romania; ^3^Department of Gastroenterology and Hepatology, “Victor Babes” University of Medicine and Pharmacy, Timisoara, Romania; ^4^Department of Biostatistics and Medical Informatics, “Victor Babes” University of Medicine and Pharmacy, Timisoara, Romania; ^5^Department of Pathology, “Victor Babes” University of Medicine and Pharmacy, Timisoara, Romania; ^6^Department of Diabetes and Metabolic Diseases, “Victor Babes” University of Medicine and Pharmacy, Timisoara, Romania

## Abstract

**Background and Aims:**

2D-shear wave elastography (2D-SWE) is a relatively new elastographic technique. The aim of the present study is to determine the values of the elasticity indexes (EI) measured by 2D-SWE in parathyroid benign lesions (adenomas or hyperplasia) and to establish if this investigation is helpful for the preoperative identification of the parathyroid adenoma.

**Material and Methods:**

The study groups were represented by 22 patients with primary or tertiary hyperparathyroidism, diagnosed by specific tests, and 43 healthy controls, in whom the thyroid parenchyma was evaluated, in order to compare the EI of the thyroid tissue with those of the parathyroid lesions.

**Results:**

The mean EI measured by 2D-SWE in the parathyroid lesions was 10.2 ± 4.9 kPa, significantly lower than that of the normal thyroid parenchyma (19.5 ± 7.6 kPa; *p* = 0.007), indicating soft tissue. For a cutoff value of 12.5 kPa, the EI assessed by 2D-SWE had a sensitivity of 93% and a specificity of 86% (AUC = 0.949; *p* < 0.001) for predicting parathyroid lesions.

**Conclusion:**

A value lower than 12.5 kPa for the mean EI measured by 2D-SWE can be used to confirm that the lesion/nodule is a parathyroid adenoma.

## 1. Introduction

Primary hyperparathyroidism has an increasing prevalence nowadays, as a consequence of routine measurements of the blood calcium levels [1, 2]. According to statistics, its main cause is represented by parathyroid adenomas [2–5]. In these cases, the curative and definitive treatment is surgery. In highly specialized centers, with skilled surgeons for this type of interventions, the localization of the adenoma is not necessary preoperatively, before first operation [6]. More than twenty-five years ago, Doppman and Miller [7] stated that “the only localization study needed in a patient with hyperparathyroidism is to localize an experienced parathyroid surgeon.” However, finding the position of the adenoma preoperatively is considered helpful for the purpose of minimally invasive surgery [8] and is mandatory before reinterventions in cases where the first surgical intervention failed [6, 9].

Imaging studies are represented by ultrasonographic (US) techniques, scintigraphy, and, rarely, computed tomography or magnetic resonance imaging. Among these, scintigraphy is considered the best, but requires a specialized center of nuclear medicine and implies an exposure to radiations [10, 11].

US is a good technique for the detection of parathyroid adenomas localized in the cervical region. In some cases, due to their location adjacent to the thyroid tissue and other cervical structures, parathyroid adenomas might be mistaken for thyroid nodules or lymph nodes.

US elastography is a noninvasive method for evaluating the mechanical characteristics of the tissues, such as elasticity and stiffness [12]. Different techniques, based on real-time two-dimensional image sequence after applying a force that is either dynamic or slowly varying (and considered “quasi-static”), were developed. The principle of elastography is based on the US measurement of tissue displacement [12, 13]. All elastographic methods use signal processing to create an image and/or to measure the stiffness and elasticity of the explored tissue.

2D-shear wave elastography (2D-SWE) uses tracking of shear wave propagation through a structure in order to evaluate the elasticity and stiffness of the tissue. The method was described in detail for the first time by Bercoff et al. in 2004 [14]. After generating the shear wave, the successive raw radiofrequency dots are acquired at a very high frame rate using an ultrafast imaging system. The velocity of the shear wave depends on the properties of the tissue, that is, it is higher in stiff structures. A color-coded image is displayed together with the B-mode picture, and quantitative information regarding the elasticity of the tissue, in terms of shear wave velocity (expressed in m/s) or estimated tissue stiffness (expressed in kilopascals (kPa)) [15], is provided.

This method was used during the past years to explore the breasts, liver, thyroid, and kidneys [16]. Parathyroid elastography was utilized in some studies with the intention to enhance the accuracy of preoperative localization of parathyroid adenomas in cases with primary hyperparathyroidism [17–19] and was found useful for minimally invasive surgery [20].

The purpose of this study was to use 2D-SWE for the evaluation of parathyroid benign lesions (adenomas or hyperplasia) in order to establish if this can offer valuable pieces of information on the preoperative localization of the adenoma.

## 2. Materials and Methods

### 2.1. Subjects

This was a cross-sectional study that included patients with hyperparathyroidism and healthy controls. Approval from the Local Ethics Committee and informed consent from all the patients and controls were obtained, before performing any study-related procedure. The research followed the Code of Ethics of the World Medical Association (Declaration of Helsinki).

The patients with hyperparathyroidism were enrolled from different outpatient or inpatient services, and 2D-SWE was offered to them after conventional US evaluation. The controls were recruited from the students and the staff of the hospital. They had a negative history for thyroid disorders and presented normal thyroid at physical and US examination.

A total number of 35 patients with hyperparathyroidism were evaluated by conventional B-mode US and by 2D-SWE. Cases with unidentified parathyroid lesion and those not submitted to surgery were excluded from the study. The remaining 22 patients formed the “parathyroid” group that included 20 patients with primary and 2 with tertiary hyperparathyroidism. The diagnosis of primary hyperparathyroidism was established in the presence of hypercalcemia with increased parathormone (PTH). The parathyroid adenoma was detected by US, and at least one supplementary imaging method (either Tc-sestamibi parathyroid scintigraphy or magnetic resonance imaging) was used for confirmation. The diagnosis of tertiary hyperparathyroidism was based on the presence of hypercalcemia, together with increased PTH, in patients known with chronic kidney failure. All the patients underwent surgery, and the pathological results confirmed the diagnosis of parathyroid adenoma or hyperplasia in the structures removed.

### 2.2. US and 2D-SWE Examination

Conventional B-mode US examination and 2D-SWE were performed with the same device—Aixplorer system (SuperSonic Imagine, France), using a high-resolution linear transducer of 15-4 MHz. B-mode US was performed first, in order to detect the pathological parathyroid and to measure the adenomatous/hyperplastic gland.

**Figure 1:**
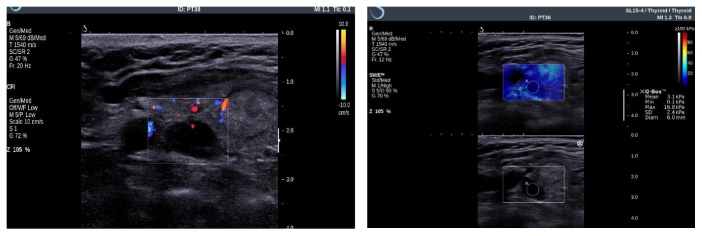
US and 2D-SWE evaluation of the parathyroid adenoma.

2D-SWE was performed in each of the cases, the image being displayed together with the grayscale US picture. After placing a box (frame) over the parathyroid adenoma captured in a longitudinal section, a colored image appeared, revealing blue and red areas on an elastogram. Dark-blue areas correspond to soft tissues, whereas red areas correspond to stiff tissues. With the aid of the device's software, a circular region of interest was placed inside the parathyroid elastogram, and the diameter of the circle was increased as much as possible, between 2 and 8 mm, taking care not to overpass the limits of the analyzed parathyroid gland (Figure 1). The default setting for thyroid 2D-SWE scale was used (range 0 to ≥100 kPa), because special settings for parathyroid exploration do not exist. The summary quantitative stiffness data were automatically displayed. The following parameters for the elasticity index (EI), expressed in kPa, were provided by the system: the mean value of the EI (SWE-Mean), the maximum value of the EI (SWE-Max), the minimum value of the EI (SWE-Min), and the standard deviation of the EI (SWE-SD). The only value that was not considered reliable, and therefore not analyzed, was the SWE-Min, because sometimes areas lacking signal could arise due to technical constraints.

We compared the EI of the abnormal parathyroid glands with that of the normal thyroid parenchyma, because normal parathyroid glands are small and cannot be evaluated by US in the majority of the subjects, despite the use of high-resolution devices.

For each parathyroid gland, three measurements were performed and the mean values for each of the three elastographic parameters depicted by the device (SWE-Mean, SWE-Max, and SWE-SD) were calculated.

For the healthy subjects from the control group, the thyroid was explored and three measurements were performed in the right thyroid lobe (RTL) and three in the left thyroid lobe (LTL). The mean value of the three determinations, for each lobe and parameter, was calculated.

All the measurements were performed by two endocrinologists (MV and IG) with more than 10 years of experience in thyroid and parathyroid US and two years of experience in using the Aixplorer device.

### 2.3. Statistical Analysis

The results were collected in a Microsoft Excel file. The statistical analysis was performed with the aid of two programs, SPSS v17 and EpiInfo v7. Descriptive statistics have been performed on both groups of subjects. The significance of the differences between groups was assessed with the Mann–Whitney test or the chi-square test. The diagnostic performance of 2D-SWE was evaluated using receiver operating characteristic curves. This analysis was performed for SWE-Mean, and the value that provided the maximum sum of sensitivity and specificity was considered the cutoff. Sensitivity and specificity were calculated according to the standard methods. The threshold for the statistical significance for *p* was set at 0.05.

## 3. Results

In total, 65 subjects were evaluated by 2D-SWE. From these, 22 patients (33.8%) were diagnosed with hyperparathyroidism (20 with primary hyperparathyroidism and 2 with tertiary hyperparathyroidism) and 43 (66.2%) were healthy controls (with normal thyroid parenchyma at US). The baseline characteristics of the cases included in the study are summarized in Table 1.

All the patients with hyperparathyroidism were submitted to surgery, and the pathological results indicated adenoma in 21 patients and hyperplasia in one case.

The mean values of EI in parathyroid adenomas and in normal thyroid parenchyma, as well as the differences between the groups, are shown in Table 2.

**Figure 2:**
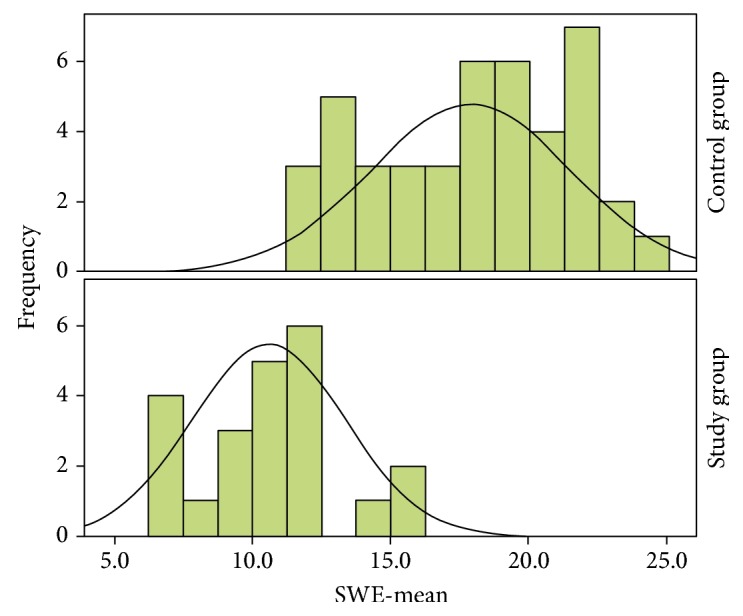
Distribution of SWE-mean for EI in healthy thyroid parenchyma and in parathyroid adenomas. SWE-Mean = mean value of the elasticity index.

The thyroid parenchyma has higher EI than the parathyroid pathological tissue, for all the parameters analyzed: SWE-Mean (Figure 2), SWE-SD, and SWE-Max.

**Figure 3:**
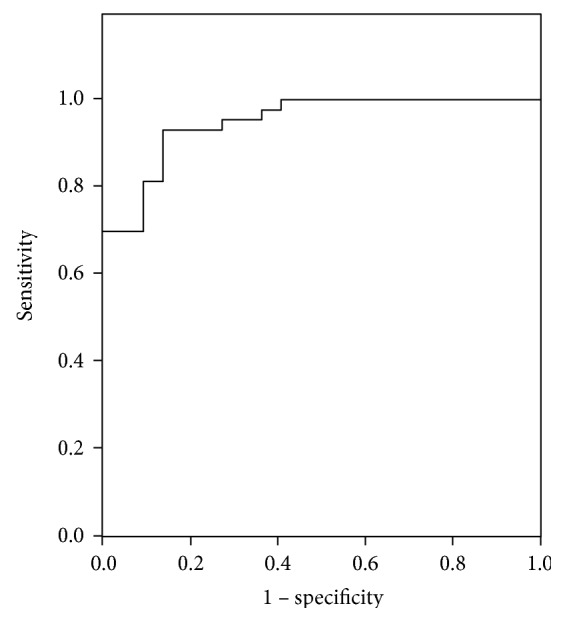
AUC for the prediction of parathyroid pathology using 2D-SWE.

The best cutoff value for predicting parathyroid pathology by SWE-Mean was calculated, and the value obtained was 12.5 kPa (AUC = 0.949; *p* < 0.001; 93% sensitivity, 86% specificity) (Figure 3), indicating that values under this cutoff point predict a parathyroid tissue.

## 4. Discussion

Elastography is a method that provides noninvasive diagnostic information regarding the elasticity of different tissues. 2D-SWE is a relatively new elastographic technique, with very good reproducibility. A correlation coefficient ranging from 0.97 to 0.98 for interobserver variability, and between 0.78 and 0.85 for intraobserver variability, was found by some authors [21].

Only few preliminary studies have used elastography in patients with parathyroid adenomas or hyperplasia, but the results are promising [17, 19, 22]. This technique was rarely applied in exploring parathyroid pathology, probably because normal parathyroid glands are too small to be explored in healthy subjects.

In our study, we analyzed the 2D-SWE characteristics of pathologically confirmed parathyroid adenomas from patients with hyperparathyroidism, in order to determine the values for the EI in abnormal parathyroid glands. We presume that knowing the EI for a specific structure would help to identify it. To the best of our knowledge, this is the first study that quantifies parameters provided by 2D-SWE with Aixplorer system in pathologically confirmed parathyroid adenomas.

Because parathyroid adenomas are usually located in the proximity of the thyroid, we utilized for comparison normal thyroid parenchyma. Our experience regarding macroscopic examination of adenomatous and hyperplasic parathyroid after surgical removal, as well as the results from two previous studies, indicates that parathyroid lesions have a soft appearance [17, 22]. These data are confirmed by our elastographic results that showed lower EI in parathyroid adenomas as compared to the thyroid tissue, the differences being significant for two of the parameters that could be measured—SWE-Mean and SWE-Max. However, one of the first studies that analyzed parathyroid lesions by the aid of elastography found that parathyroid adenomas were quite stiff [19]. The total discordance with our results could be explained by the different elastographic techniques used: 2D-SWE in our case and strain elastography in the aforementioned study.

The values of the EI in the two thyroid lobes in healthy subjects are similar [23], so that the evaluation of one of them is sufficient for the comparison with the pathologic parathyroid gland.

We suggest that, for predicting parathyroid adenomas, the cutoff value for SWE-Mean should be set at 12.5 kPa. This provides the maximum sum of sensitivity and specificity for the measurement and a minimum probability for having false results. Subjects with a SWE-Mean value smaller than 12.5 kPa have, with a very high probability, a parathyroid adenoma and need no additional imagistic tests to localize it. These values are very useful when elastography is used to confirm that the nodule detected by US represents a pathological parathyroid gland.

In clinical practice, parathyroid adenomas need to be differentiated by other neck lesions, such as thyroid nodules and lymph nodes. Two studies, using Virtual Touch Imaging Quantification, performed a direct comparison between parathyroid and thyroid lesions, but their results are not concordant. Chandramohan et al. [22] found that parathyroid adenomas were softer than benign and malignant thyroid nodules. Batur et al. [20] reported that the stiffness of parathyroid adenomas was higher than that of benign, but lower than that of malignant thyroid nodules. The values of EI in parathyroid adenomas found in our study are lower than the ones reported by different authors for benign thyroid nodules, using the same elastographic technique: 35.1 ± 30.6 kPa [24], 41.0 ± 25.8 kPa [25], and 51.4 ± 22.7 kPa [26]. In all of the aforementioned studies, the malignant thyroid nodules had a much higher stiffness.

Sometimes, it may be quite challenging to differentiate parathyroid adenomas from cervical lymph nodes. Two recent studies, using a different elastographic technique [17, 27], reported that parathyroid adenomas have a lower shear wave velocity in comparison to benign lymph nodes. 2D-SWE with the Aixplorer system was used by some authors to characterize cervical lymphadenopathies. The values obtained by them in benign lymph nodes were 14.2 ± 4.1 kPa [28], 27.5 ± 18.9 kPa [29], and 23.3 ± 25.3 kPa [30]. In all the studies, malignant lymph nodes were significantly stiffer.

Considering all these data, one can state that parathyroid adenomas are softer than thyroid nodules and lymphadenopathies by 2D-SWE with the Aixplorer system.

The technique proved to have a good feasibility, so it can be offered after routine US examination in the cases where a parathyroid adenoma is suspected.

There are several limitations of our study. First, the number of cases with hyperparathyroidism is low. However, all of them were treated by surgery, so that there is confirmation provided by the pathologic exam for all the parathyroid lesions. Second, the selection of the subjects with normal thyroid was based only on a negative history for thyroid disorders, normal physical exam (performed by an experienced endocrinologist), and normal thyroid US examination. No hormonal or immunological measurements were performed. Nevertheless, we considered these criteria sufficient to exclude, with a high probability, any thyroid pathology. Third, because normal parathyroid tissue cannot be evaluated in the majority of the subjects, the values for EI in parathyroid adenomas were compared with those of normal thyroid tissue. We do not consider that this last limitation constituted an important bias, because our intention was to establish a cutoff value for EI that could be used as a threshold when analyzing parathyroid adenomas and to distinguish these pathological parathyroid glands from the nearby normal thyroid parenchyma.

The work performed by us has several strengths, as well. To the best of our knowledge, this is the first study that used this elastographic method to investigate the parathyroid glands. In addition, due to the fact that the parathyroids were surgically removed, they could be evaluated by the pathologist, providing the confirmation that all the analyzed lesions represent parathyroid tissue. Finally, a standard protocol was used in all cases by two investigators with a lot of experience in using 2D-SWE.

Further studies are needed in order to compare the different parameters that can be measured by 2D-SWE for different parathyroid lesions with those obtained in thyroid nodules and/or cervical lymph nodes.

To conclude, in this study, we intended to quantify the values of EI measured by 2D-SWE, in order to provide a tool to identify preoperatively the presence of a parathyroid lesion, when other imagistic methods are not available. 2D-SWE can conveniently be performed in routine clinical practice, after US examination, in patients with parathyroid adenomas. The values of different EI measured by 2D-SWE in parathyroid adenomas are significantly lower than those of the normal thyroid parenchyma, indicating the existence of a soft tissue. By using this elastographic technique, a value less than 12.5 kPa for mean EI could be used to confirm that the lesion/nodule is a parathyroid adenoma.

## Figures and Tables

**Table 1:** Baseline characteristics of the studied cases.

Parameter	Study group	Controls	*p*
Number^a^	22	43	—
F/M^a^	19/3	30/13	—
Age (years)^b^	53.7 ± 13.6	48.3 ± 11.3	0.887
Weight (kg)^b^	73.7 ± 18.6	66.3 ± 17.5	0.166
TV (ml)^b^	—	9.3 ± 3.3	—
Calcemia (NV: 8.2–10.4 mg/dl)^b^	10.8 ± 1.5	—	—
PTH (NV: 10–55 pg/ml)^b^	411.5 ± 594.1	—	—
Maximum diameter of PT adenoma in US (mm)^b^	14.3 ± 6.4	—	—

F: females; M: males; TV: thyroid volume; NV: normal values; PTH: parathormone; PT: parathyroid; ^a^values expressed as numbers; ^b^values expressed as mean ± standard deviation.

**Table 2:** Mean values of 2D-SWE parameters for parathyroid and thyroid tissue, in the two groups of subjects.

Samples/variables	SWE-Mean (kPa)	SWE-Max (kPa)	SWE-SD (kPa)
Control group			
RTL^a^	19.6 ± 6.6	33.4 ± 9.5	4.1 ± 1.4
LTL^a^	19.5 ± 6.8	33.2 ± 11.2	4.4 ± 1.7
*p* (RTL versus LTL)	0.92	0.91	0.29
Mean RTL-LTL^a^	19.5 ± 7.6	33.4 ± 9.5	4.3 ± 1.5
Study group^a^	10.2 ± 4.9	22.9 ± 10.6	4.8 ± 2.5
*p* (RTL versus parathyroid adenoma)	<0.001	<0.001	0.233
*p* (mean RTL-LTL versus parathyroid adenoma)	<0.001	<0.001	0.396

SWE: shear wave elastography; RTL: right thyroid lobe; LTL: left thyroid lobe; mean: mean value; max: maximum value; SD: standard deviation; ^a^values expressed as mean ± standard deviation.

## References

[1] Hindié E., Ugur O., Fuster D. (2009). 2009 EANM parathyroid guidelines. *European Journal of Nuclear Medicine and Molecular Imaging*.

[2] Bilezikian J. P., Brandi M. L., Eastell R. (2014). Guidelines for the management of asymptomatic primary hyperparathyroidism: summary statement from the Fourth International Workshop. *The Journal of Clinical Endocrinology and Metabolism*.

[3] Johnson N. A., Tublin M. E., Ogilvie J. B. (2007). Parathyroid imaging: technique and role in the preoperative evaluation of primary hyperparathyroidism. *AJR American Journal of Roentgenology*.

[4] Pyram R., Mahajan G., Gliwa A. (2011). Primary hyperparathyroidism: skeletal and non-skeletal effects, diagnosis and management. *Maturitas*.

[5] Marcocci C., Bollerslev J., Khan A. A., Shoback D. M. (2014). Medical management of primary hyperparathyroidism: proceedings of the Fourth International Workshop on the management of asymptomatic primary hyperparathyroidism. *The Journal of Clinical Endocrinology and Metabolism*.

[6] Wilhelm S. M., Wang T. S., Ruan D. T. (2016). The American Association of Endocrine Surgeons guidelines for definitive management of primary hyperparathyroidism. *JAMA Surgery*.

[7] Doppman J. L., Miller D. L. (1991). Localization of parathyroid tumors in patients with asymptomatic hyperparathyroidism and no previous surgery. *Journal of Bone and Mineral Research*.

[8] Patel C. N., Salahudeen H. M., Lansdown M., Scarsbrook A. F. (2010). Clinical utility of ultrasound and 99mTc sestamibi SPECT/CT for preoperative localization of parathyroid adenoma in patients with primary hyperparathyroidism. *Clinical Radiology*.

[9] Nieciecki M., Cacko M., Królicki L. (2015). The role of ultrasound and nuclear medicine methods in the preoperative diagnostics of primary hyperparathyroidism. *Journal of Ultrasonography*.

[10] De Feo M. L., Colagrande S., Biagini C. (2000). Parathyroid glands: combination of (99m)Tc MIBI scintigraphy and US for demonstration of parathyroid glands and nodules. *Radiology*.

[11] Casara D., Rubello D., Piotto A., Pelizzo M. R. (2000). 99mTc-MIBI radio-guided minimally invasive parathyroid surgery planned on the basis of a preoperative combined 99mTc-pertechnetate/99mTc-MIBI and ultrasound imaging protocol. *European Journal of Nuclear Medicine*.

[12] Bamber J., Cosgrove D., Dietrich C. F. (2013). EFSUMB guidelines and recommendations on the clinical use of ultrasound elastography. Part 1: basic principles and technology. *Ultraschall in der Medizin*.

[13] Gennisson J. L., Deffieux T., Fink M., Tanter M. (2013). Ultrasound elastography: principles and techniques. *Diagnostic and Interventional Imaging*.

[14] Bercoff J., Tanter M., Fink M. (2004). Supersonic shear imaging: a new technique for soft tissue elasticity mapping. *IEEE Transactions on Ultrasonics, Ferroelectrics, and Frequency Control*.

[15] Sebag F., Vaillant-Lombard J., Berbis J. (2010). Shear wave elastography: a new ultrasound imaging mode for the differential diagnosis of benign and malignant thyroid nodules. *The Journal of Clinical Endocrinology and Metabolism*.

[16] Shiina T., Nightingale K. R., Palmeri M. L. (2015). WFUMB guidelines and recommendations for clinical use of ultrasound elastography: part 1: basic principles and terminology. *Ultrasound in Medicine & Biology*.

[17] Azizi G., Piper K., Keller J. M. (2016). Shear wave elastography and parathyroid adenoma: a new tool for diagnosing parathyroid adenomas. *European Journal of Radiology*.

[18] Hattapoğlu S., Göya C., Hamidi C. (2016). Evaluation of parathyroid lesions with point shear wave elastography. *Journal of Ultrasound in Medicine*.

[19] Ünlütürk U., Erdoğan M. F., Demir O., Culha C., Güllü S., Başkal N. (2012). The role of ultrasound elastography in preoperative localization of parathyroid lesions: a new assisting method to preoperative parathyroid ultrasonography. *Clinical Endocrinology*.

[20] Batur A., Atmaca M., Yavuz A. (2016). Ultrasound elastography for distinction between parathyroid adenomas and thyroid nodules. *Journal of Ultrasound in Medicine*.

[21] Bhatia K., Tong C. S., Cho C. C., Yuen E. H., Lee J., Ahuja A. T. (2012). Reliability of shear wave ultrasound elastography for neck lesions identified in routine clinical practice. *Ultraschall in der Medizin*.

[22] Chandramohan A., Therese M., Abhraham D., Paul T. V., Mazhuvanchary P. J. (2017). Can ARFI elastography be used to differentiate parathyroid from thyroid lesions?. *Journal of Endocrinological Investigation*.

[23] Vlad M., Golu I., Bota S. (2015). Real-time shear wave elastography may predict autoimmune thyroid disease. *Wiener Klinische Wochenschrift*.

[24] Szczepanek-Parulska E., Woliński K., Stangierski A. (2013). Comparison of diagnostic value of conventional ultrasonography and shear wave elastography in the prediction of thyroid lesions malignancy. *PLoS One*.

[25] Veyrieres J. B., Albarel F., Lombard J. V. (2012). A threshold value in shear wave elastography to rule out malignant thyroid nodules: a reality?. *European Journal of Radiology*.

[26] Kim H., Kim J. A., Son E. J., Youk J. H. (2013). Quantitative assessment of shear-wave ultrasound elastography in thyroid nodules: diagnostic performance for predicting malignancy. *European Radiology*.

[27] Cheng K. L., Choi Y. J., Shim W. H., Lee J. H., Baek J. H. (2016). Virtual touch tissue imaging quantification shear wave elastography: prospective assessment of cervical lymph nodes. *Ultrasound in Medicine & Biology*.

[28] Choi Y. J., Lee J. H., Lim H. K. (2013). Quantitative shear wave elastography in the evaluation of metastatic cervical lymph nodes. *Ultrasound in Medicine & Biology*.

[29] Jung W. S., Kim J. A., Son E. J., Youk J. H., Park C. S. (2015). Shear wave elastography in evaluation of cervical lymph node metastasis of papillary thyroid carcinoma: elasticity index as a prognostic implication. *Annals of Surgical Oncology*.

[30] Desmots F., Fakhry N., Mancini J. (2016). Shear wave elastography in head and neck lymph node assessment: image quality and diagnostic impact compared with B-mode and Doppler ultrasonography. *Ultrasound in Medicine & Biology*.

